# Evolution of *Caenorhabditis elegans* host defense under selection by the bacterial parasite *Serratia marcescens*

**DOI:** 10.1371/journal.pone.0181913

**Published:** 2017-08-09

**Authors:** McKenna J. Penley, Giang T. Ha, Levi T. Morran

**Affiliations:** Department of Biology, Emory University, Atlanta, Georgia, United States of America; Centre National de la Recherche Scientifique & University of Nice Sophia-Antipolis, FRANCE

## Abstract

Parasites can impose strong selection on hosts. In response, some host populations have adapted via the evolution of defenses that prevent or impede infection by parasites. However, host populations have also evolved life history shifts that maximize host fitness despite infection. Outcrossing and self-fertilization can have contrasting effects on evolutionary trajectories of host populations. While selfing and outcrossing are known to affect the rate at which host populations adapt in response to parasites, these mating systems may also influence the specific traits that underlie adaptation to parasites. Here, we determined the role of evolved host defense versus altered life history,in mixed mating (selfing and outcrossing) and obligately outcrossing *C*. *elegans* host populations after experimental evolution with the bacterial parasite, *S*. *marcescens*. Similar to previous studies, we found that both mixed mating and obligately outcrossing host populations adapted to *S*. *marcescens* exposure, and that the obligately outcrossing populations exhibited the greatest rates of adaptation. Regardless of the host population mating system, exposure to parasites did not significantly alter reproductive timing or total fecundity over the course of experimental evolution. However, both mixed mating and obligately outcrossing host populations exhibited significantly reduced mortality rates in the presence of the parasite after experimental evolution. Therefore, adaptation in both the mixed mating and obligately outcrossing populations was driven, at least in part, by the evolution of increased host defense and not changes in host life history. Thus, the host mating system altered the rate of adaptation, but not the nature of adaptive change in the host populations.

## Introduction

Parasites are prevalent in nature and capable of imposing significant fitness costs on their hosts. Consequently, host populations have evolved numerous strategies for mitigating or preventing infection by parasites [1, 2]. Hosts commonly adapt via the evolution of novel or enhanced forms of defense in response to parasites. The specific traits and mechanisms that underlie host defense can vary greatly, from heighted cell-mediated immunity to recognition and avoidance of the parasite upon exposure [3]. As opposed to an inducible defense strategy, some host populations have instead evolved altered life histories [4–6]. Indeed, after evolving under frequent threat of infection, some host populations exhibit reduced times to sexual maturity and earlier reproduction as a means of maximizing fitness [7, 8]. Thus, the nature of host adaptation to parasites can vary greatly, as hosts employ many different mechanisms of combating fitness loss due to parasite infection.

Many factors may influence the evolutionary trajectories of host populations in response to selection imposed by parasites [9]. The host mating system can determine whether host populations respond sufficiently to selection from coevolving parasites or are driven to extinction [10]. Further, self-fertilization and outcrossing, different forms of sexual reproduction, can have very different effects on the evolutionary trajectories of populations in response to natural selection [11–19]. In general, outcrossing can facilitate more rapid adaptation to selection than self-fertilization by accelerating the breakdown of linkage disequilibrium within populations [20–22]. Further, breaking genetic linkage can permit the assembly of novel beneficial allelic combinations [23, 24] and disassociate beneficial mutations from deleterious mutations [21, 22, 25–27]. Additionally, the propensity of selfing to generate and maintain linkage disequilibrium can slow adaptation due to selective interference from deleterious mutations at loci in close proximity to beneficial alleles. Overall, adaptation via outcrossing may result in the fixation of fewer deleterious alleles and incorporate a greater number of loci relative to self-fertilization. By increasing the efficacy of selection, outcrossing may also permit selection to act on a greater number of traits simultaneously, whereas adaptation in selfing populations may often be restricted to the trait with the greatest contribution to fitness [25–28]. Therefore, the host mating system may play a prominent role in determining the mechanisms underlying host adaptation to parasites. Nonetheless, it is generally unclear how selfing and outcrossing may affect the mechanisms and specific traits that contribute to host adaptation in response to selection imposed by parasites

*Caenorhabditis elegans* are androdioecious nematodes that reproduce via self-fertilization and outcrossing (mixed mating) [29]. However, *C*. *elegans* populations harboring the fog-2(q7) mutant allele, which disables sperm production in hermaphrodites, are obligately outcrossing [30]. Conversely, *C*. *elegans* populations carrying the xol-1(y9) mutation, which is lethal in males, are obligately selfing [31]. *Serratia marcescens* is a virulent bacterial parasite that can kill *C*. *elegans* hosts when consumed [32]. Previous host-parasite experimental evolution studies, in which hosts were consistently exposed to the same genotype of *S*. *marcescens* each generation, found that both obligately outcrossing and mixed mating *C*. *elegans* populations rapidly adapted to *S*. *marcescens*. However, obligately selfing populations did not adapt to the parasites [10, 20, 33–35], and the obligately outcrossing populations exhibited greater increases in fitness relative to the mixed mating populations [20, 34]. Therefore, high levels of outcrossing facilitated greater rates of adaptation to the parasites. Indeed, greater rates of outcrossing evolved in mixed mating host populations, indicating that outcrossing was favored by selection imposed by the parasite [10, 20, 33].

Apart from different rates of adaptive change, it is unclear if mixed mating can alter the evolutionary trajectories of host populations relative to obligate outcrossing. In a previous experiment, hosts were required to survive exposure to *S*. *marcescens* strain Sm2170, locate and consume their *Escherichia coli* food source, then reproduce before experimental passage [10]. Within the context of the experimental system, host fitness was determined by both survival in the presence of the parasite and fecundity after parasite exposure. The host populations may have adapted to the parasite and the selection regime through increased host defense and/or the evolution of greater or earlier reproduction. Here, we tested for adaptation in these mixed mating and obligately outcrossing host populations that were passaged either in the presence of live *S*. *marcescens* Sm2170 or heat-killed *S*. *marcescens*, as a control, for thirty generations [10]. We then investigated the evolutionary trajectories of the host populations by linking changes in fitness with changes in host defense versus shifts in reproductive timing.

## Materials and methods

### Experimental host populations

Experimental host populations consisted of 5 replicates of mixed mating *C*. *elegans* strain PX382 and five replicates of obligately outcrossing *C*. *elegans* strain PX386, both from an overall CB4856 background [20], which was originally obtained from the Caenorhabditis Genetics Center. Strain PX386 harbors the fog-2(q71) mutation which prevents hermaphrodites from producing sperm, therefore creating an obligately outcrossing population. All experimental populations were independently mutagenized with Ethyl Methanesulfonate (EMS) to introduce novel genetic variation prior to selection [10]. The ancestor to each experimental population was frozen and stored at -80°C prior to experimental evolution, while the experimental populations were frozen after 30 generations of experimental evolution. Each of the ten replicate populations (five mixed mating and five obligately outcrossing) was then replicated across each treatment, such that a sample of each replicate population underwent each treatment.

### Experimental evolution

All populations underwent 30 generations of passage on Serratia Selection Plates (SSP) as described in in a previous study [10]. Briefly, *S*. *marcescens* strain Sm2170, obtained from the lab of Curtis Lively at Indiana University, was grown on approximately 1/3 of a Petri dish, OP50 *E*. *coli* on the opposite 1/3 of the plate, with ampicillin (200 μg/mL) spread in the middle 1/3 of the plate. *C*. *elegans* are plated on the *S*. *marcescens* lawn and must survive exposure to the parasite and reproduce on the OP50 lawn to have measurable fitness during the experiment. Five genetically independent mixed mating and five genetically independent obligately outcrossing populations were exposed to 2 different treatments, (Evolution) exposure to a fixed strain of *S*. *marcescens* Sm2170, and (Control) exposure to heat-killed Sm2170. SSPs were constructed using NGM Lite (US Biological, Swampscott, MA) and stored at 20°C throughout the experiment. *C*. *elegans* host populations were transferred every generation following methods outlined in the previous study [10].

### Change in fitness

Change in fitness after 30 generations of experimental evolution was measured using Competitive Fitness Assays (CFA) [20]. CFAs were conducted for the ancestral populations and experimental populations, all of which were thawed after storage at -80°C, using methods from [10]. Briefly, 100 experimental or ancestral individuals and 100 GFP labeled tester strain individuals (strain JK2735) were placed on SSPs with live *S*. *marcescens*. Individuals were permitted eat, grow, and reproduce. Then, we measured the ratio of experimental or ancestral population offspring to tester strain offspring after one generation [20]. At least two replicate CFAs were conducted for each host population. Importantly, the GFP marker is dominant, therefore the offspring of outcrossing between experimental males and GFP-labeled hermaphrodites (all tester strain individuals were hermaphrodites) appear as tester strain offspring. Given that the obligately outcrossing host populations harbor significantly greater numbers of males, relative to mixed mating populations, the competitive fitness measurements of the obligately outcrossing populations are more conservative estimates. We performed a Shapiro-Wilk test and Levene’s test, and found that the data did not violate assumptions of normality nor homogeneity of variance. Then we performed an ANOVA in JMP 12 (SAS Institute, Cary, NC) testing the effects of host mating system (mixed mating versus obligately outcrossing), treatment (Ancestor, Control, Evolution), host replicate population (treated as a random factor nested within mating system), and the interaction of mating system and treatment on the mean ratio of experimental to tester strain offspring. Further, we conducted least squares mean linear contrast tests within the mixed mating and obligately outcrossing populations contrasting the means of Evolution treatment with the means of Control and Ancestral populations. Lastly, we performed a Kruskal Wallis test to analyze the effects of host mating system on the change in fitness relative to the ancestral population. The change in host fitness relative to the ancestral population was calculated as described in [20]. Bonferroni correction was applied to this data set due to multiple statistical tests (P < 0.025).

### Host defense

Host defense was measured using Survival Assays (SA), wherein 200 *C*. *elegans* from the either an ancestral or experimental (after 30 generations of selection) population were exposed to live Sm2170 on SSPs and scored for survival after 48 hours. We calculated the mean mortality rates for each experimental and ancestral population, running at least 3 replicate assays for each population. We chose a 48 hour time period because Sm2170 rapidly kills infected *C*. *elegans*, often within 24 hours of consumption. *C*. *elegans* hosts move off the Sm2170 lawn and onto OP50 within the first 24 hours of exposure to the SSP. Further, *C*. *elegans* hosts must reproduce within the first 48 hours of exposure on the SSP to ensure that their offspring are passaged to the next generation. So, measuring rates of mortality after 48 hours of exposure is an indicator of the number of individuals that survived to reproductive age in the context of our experiment. We performed Shapiro-Wilk and Levene’s tests, and found that the data did not violate assumptions of ANOVA. We then performed an ANOVA in JMP 12 testing the effects of host mating system, treatment, host replicate population (treated as a random factor nested within mating system), and the interaction of mating system and treatment on the mean mortality rates calculated for each ancestral and experimental population. Further, we conducted least squares mean linear contrast tests within treatment to test the effects of individual treatments (Evolution versus Control and Evolution versus Ancestral populations).

### Host fecundity

Host fecundity was calculated for both the ancestral and experimental populations (after 30 generations of selection) by measuring reproduction over seven days, in the absence of the parasite, beginning at the L4 lifestage of the hermaphrodite or female. For obligately outcrossing populations, L4 female offspring were picked onto 35mm plates seeded with 20μl of OP50, along with two adult males from the female’s respective replicate population. All three worms were passaged together throughout the assay to permit further reproduction. For mixed mating populations, one hermaphrodite per plate, starting at the L4 lifestage, was passaged throughout the assay. Importantly, fecundity was assessed using the predominant mating system employed by the populations after 30 generations of experimental evolution. Specifically, the obligately outcrossing populations reproduced via obligate outcrossing, whereas most offspring (~80%) in the mixed mating populations were produced via selfing [10]. *C*. *elegans* were transferred to a new 35mm plate at 48 hours after the initial plating. Then focal individuals were again transferred after a subsequent 36 hours and 120 hours from the beginning of the assay to clearly delineate between focal parents and offspring, and to assess reproductive timing. We conducted this assay for five replicate females or hermaphrodites from each replicate population. The quantity of L1 offspring produced was measured after each passage and on day seven, and the mean lifetime fecundity and 48-hour fecundity was calculated for each replicate population. For both the mean lifetime and mean 48-hour fecundity data sets, we found that the data did not violate the assumptions of ANOVA using Shapiro-Wilk and Levene’s tests. Then, we performed an ANOVA in JMP 12 testing the effects of host mating system, treatment, host replicate population (treated as a random factor nested within mating system), and the interaction of mating system and treatment on the mean fecundity calculated for each ancestral and experimental population. We also conducted least squares mean linear contrast tests within treatment to test the effects of individual treatments (Control versus Evolution and Control versus Ancestral populations). Bonferroni correction was applied to this data due to multiple statistical tests (P < 0.025).

## Results

### Change in fitness

**Fig 1:**
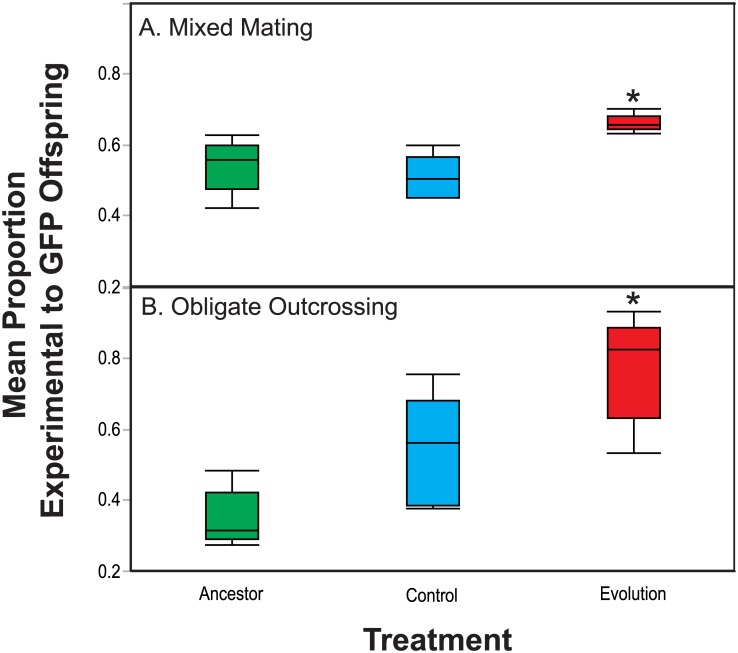
Competitive fitness during exposure to *S*. *marcescens*. Populations exposed to the Control and Evolution treatments, as well their Ancestral populations, were competed against a tester strain in the presence of *S*. *marcescens*. Experimental and tester strain individuals were exposed to the parasite, starting at a 1:1 ratio, for one generation. Greater mean proportions of experimental individuals indicate greater competitive fitness. (A) Mixed mating populations exposed to the Evolution treatment exhibit greater competitive fitness than the Ancestral or Control populations. (B) Obligately outcrossing populations exposed to the Evolution treatment exhibit greater competitive fitness than their respective Ancestral and Control populations.

After thirty generations of exposure to the same strain of *S*. *marcescens* in the Evolution treatment, both the mixed mating and obligately outcrossing populations exhibited increased competitive fitness in the presence of the parasite, relative to the Control and Ancestral populations (Fig 1A and Table 1; Mixed mating: Evolution vs Control; F_1,16_ = 8.25, P = 0. 011, Evolution vs Ancestor; F_1,16_ = 4.70, P = 0. 045) (Fig 1B and Table 1; Obligate outcrossing: Evolution vs Control; F_1,16_ = 18.14, P = 0.0006, Evolution vs Ancestor; F_1,16_ = 59.64, P < 0. 0001). Therefore, both the mixed mating and obligately outcrossing populations adapted to *S*. *marcescens* exposure. However, the rate of adaptation differed between mating systems. The obligately outcrossing populations adapted to *S*. *marcescens* at a greater rate than the mixed mating populations. Specifically, the obligately outcrossing host populations in the Evolution treatment exhibited a significantly greater increase in fitness relative to their ancestral populations, than did the mixed mating hosts populations (Obligate outcrossing increase in fitness relative to ancestor = 131%, mixed mating increase in fitness relative to ancestor = 24%; χ^2^_1_ = 5.771, P = 0.0163). However, it should be noted that the competitive fitness of the obligately outcrossing ancestral populations was lower than the fitness of the ancestral mixed mating populations (Fig 1), which contributed to the difference in the rates of adaptation between mating systems.

**Table 1:** ANOVA table for competitive fitness.

Source	Sum of Squares	df	Mean Square	F	P
Model	0.66	13	0.05	6.69	0.0003
Error	0.12	16	0.007		
Total	0.78	29			
Mating	0.002	1	.002	0.14	0.722
Treatment	0.394	2	0.197	25.95	< 0.0001
Population (Mating)	0.138	8	0.017	2.27	0.077
Mating x Treatment	0.126	2	0.063	8.29	0.003

### Host defense

**Fig 2:**
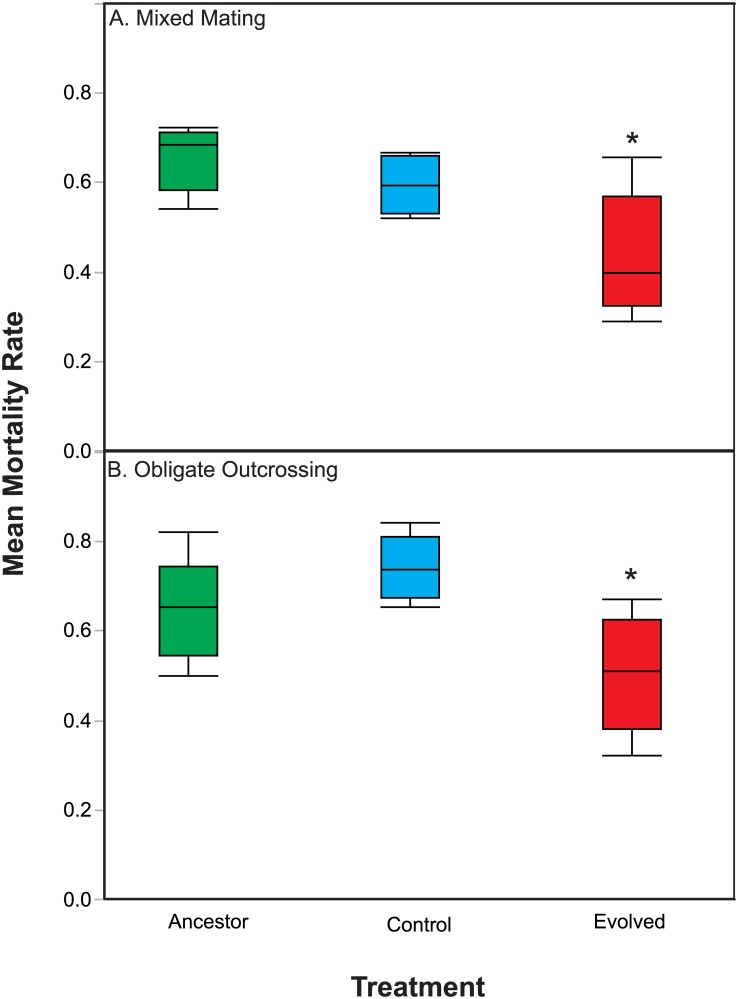
Host defense during exposure to *S*. *marcescens*. Populations evolved under the Control and Evolution treatments, as well as Ancestral populations, were exposed to *S*. *marcescens* on SSPs for 48 hours and then host mortality rates were assessed. (A) Mixed mating populations from the Evolution treatment exhibited reduced mortality rates. (B) Obligately outcrossing populations from the Evolution treatment exhibited reduced mortality rates. Overall, persistent exposure to the parasite selected for the evolution of increased host defense regardless of the host mating system.

The increased fitness in the presence of *S*. *marcescens* exhibited by *C*. *elegans* populations in the Evolution treatment could be the result of increased levels of defense against the parasite, a life history shift resulting in increased host fecundity, or a combination of both changes. We assessed changes in host defense by exposing Control, Evolution, and Ancestral populations to *S*. *marcescens* on SSPs and we measured rates of host mortality over 48 hours. Overall, we found a significant treatment effect (Table 2), in the mixed mating and obligately outcrossing populations from the Evolution treatment exhibiting significantly reduced mortality rates relative to Control (Fig 2 and Table 2; F_1,16_ = 20.69 P = 0. 0003) and Ancestral populations (Fig 2 and Table 2; F_1,16_ = 17.20, P = 0.0008). Therefore, adaptation in both the mixed mating and obligately outcrossing populations was at least partially driven by increased host defense.

**Table 2:** ANOVA table for mean host mortality rates.

Source	Sum of Squares	df	Mean Square	F	P
Model	0.599	13	0.046	3.50	0.01
Error	0.211	16	0.013		
Total	0.81	29			
Mating	0.062	1	0.062	3.11	0.1156
Treatment	0.335	2	0.168	12.68	0.0005
Population (Mating)	0.16	8	0.02	1.52	0.227
Mating x Treatment	0.042	2	0.021	1.61	0.23

### Host fecundity

**Fig 3:**
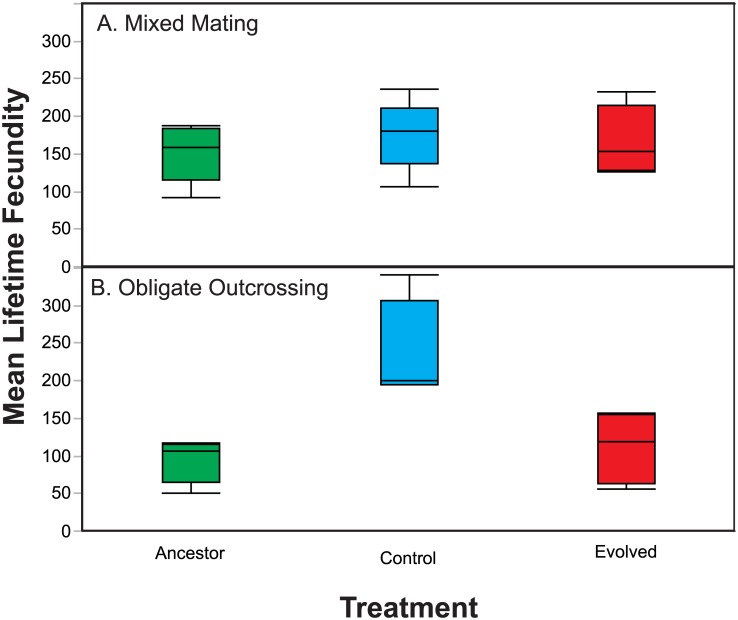
Host lifetime fecundity. Lifetime fecundity was assessed in the absence of *S*. *marcescens* for Control, Evolution, and Ancestral populations in the (A) mixed mating populations and the (B) obligately outcrossing populations. Control populations exhibited greater levels of fecundity than the Ancestral and Evolution populations in obligately outcrossing populations, but no treatment differences were detected in the mixed mating populations.

We assessed changes in host fecundity over the course of experimental evolution as a potential mechanism contributing to adaptation. Specifically, we measured the lifetime fecundity, in the absence of the parasite, for populations that evolved under the Control and Evolution treatments, as well as the Ancestral populations. Overall, we did not observe a treatment effect in the mixed mating populations as the Control and Evolution populations did not exhibit significantly different levels of fecundity (Fig 3A and Table 3; F_1,14_ = 0.077, P = 0.785) However, we found that the mean lifetime fecundity increased in the obligately outcrossing Control populations relative to the Evolution (Fig 3B and Table 3; F_1,14_ = 18.49, P = 0.0007) and Ancestral (Fig 3B and Table 3; F_1,14_ = 26.11, P = 0.0002) populations. Nonetheless, the Control populations did not adapt to *S*. *marcescens* exposure (Fig 1), whereas the populations exposed to the Evolution treatment exhibited adaptive change. Therefore the adaptive change in the Evolution populations cannot be attributed to increases in host fecundity in the absence of the parasite.

**Table 3:** ANOVA table for mean lifetime fecundity.

Source	Sum of Squares	df	Mean Square	F	P
Model	77793	13	5984	3.31	0.017
Error	25288	14	1806		
Total	103082	27			
Mating	2166	1	2166	0.74	0.414
Treatment	37843	2	18921	10.47	0.002
Population (Mating)	23564	8	2945	1.63	0.202
Mating x Treatment	23432	2	11716	6.49	0.01

**Fig 4:**
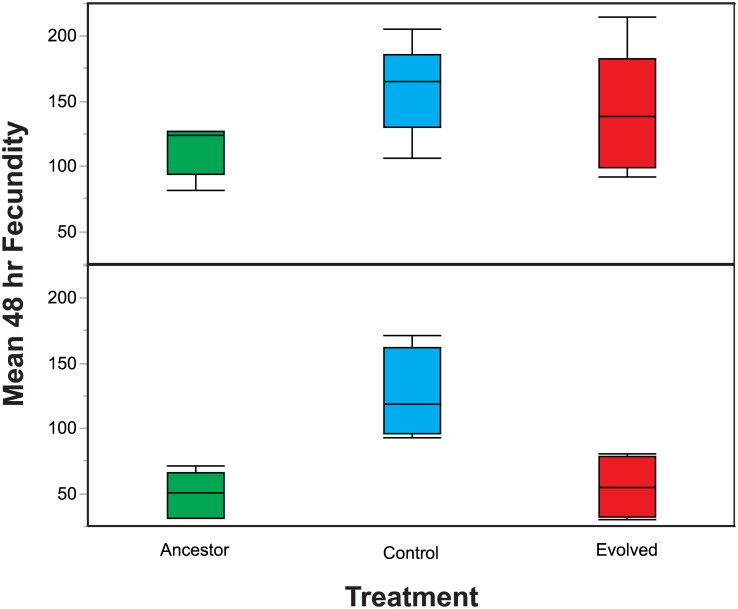
Host 48-hour fecundity. *C*. *elegans* fecundity was measured over a 48 hour period in the absence of *S*. *marcescens*. Control populations exhibited increased mean 48-hour fecundity in both the (A) mixed mating and (B) obligately outcrossing populations. Additionally, the mixed mating populations exhibited greater mean 48-hour fecundity than the obligately outcrossing populations.

Within the context of our experiment, fecundity within the first 48 hours of exposure to the SSP is likely a better measure of fitness than the host’s total lifetime fecundity. During experimental evolution, larval hosts were transferred to the SSP and then their offspring were transferred to a new SSP after 4 days. Early reproduction likely disproportionately contributed to fitness relative to lifetime fecundity, given the short time window for reproduction before subsequent transfer. Further, *S*. *marcescens* strain Sm2170 can rapidly kill infected hosts, often in less than 48 hours (10). Therefore, rapid reproduction may have been under selection in our experiment to permit reproduction despite infection. We measured the mean 48-hour fecundity of the Control, Evolution, and Ancestral populations in the absence of the parasite. Fecundity was measured in the absence of the parasite to determine the role of shifts in reproductive timing as a potential mechanism underlying changes in fitness exhibited by the experimental populations. Overall, we found significantly greater levels of mean 48-hour fecundity in the Control populations (Fig 4 and Table 4). Additionally, we found that the mixed mating populations exhibited greater 48-hour fecundity than the obligately outcrossing populations (Fig 4 and Table 4). Importantly, these early fecundity differences may be due to mate limitation as a result of reliance on male mating within the obligately outcrossing populations, whereas the mixed mating hermaphrodites self-fertilized. The observed shift in reproductive timing within the Control populations may be the result of adaptation to the experimental evolution regime. Nonetheless, whether lifetime or 48-hour fecundity, the result of increased fecundity in the Control populations does not sufficiently explain the adaptive change observed in the Evolution populations relative to the Controls.

**Table 4:** ANOVA table for mean 48-hour fecundity.

Source	Sum of Squares	df	Mean Square	F	P
Model	61294	13	4714	6.87	0.0005
Error	9609	14	686		
Total	70903	27			
Mating	25420	1	25420	15.7	0.0004
Treatment	20214	2	10107	14.7	0.004
Population (Mating)	13069	8	1633	2.38	0.075
Mating x Treatment	4260	2	2130	3.1	0.077

## Discussion

Overall, our results reinforce the results presented by Morran et al [20]. Obligately outcrossing and mixed mating populations evolved in the presence of live *S*. *marcescens* both adapted to parasite exposure over 30 generations (Fig 1). Further, the obligately outcrossing populations exhibited a greater increase in fitness relative to their ancestral populations. Nonetheless, the rate and magnitude of adaptation are only two aspects of a population’s evolutionary trajectory, and outcrossing and selfing have the potential to influence many aspects of adaptive change in populations. Given the contrasting genetic consequences of outcrossing and selfing, both the traits evolving under selection and the genetic architecture underlying adaptation may differ between mixed mating and obligately outcrossing populations.

Here, we assessed the role of increased host defense against parasites versus shifts in life history as means of characterizing adaptive evolution in the obligately outcrossing and mixed mating populations. We assayed survival in the presence of *S*. *marcescens* as a means of testing host defense, while we measured fecundity in the absence of the parasite to test for shifts in life history. Importantly, we conducted fecundity assays in the absence of the parasite, because parasite-induced fecundity is a form of host defense and we sought to assess general changes in host life history. Regardless of the host mating system, we found that host populations passaged in the presence of the live parasite evolved increased defense to the parasite, resulting in decreased mortality in the presence of the parasite in comparison to ancestral populations and populations passaged with heat-killed parasite (Fig 2). With regards to changes in fecundity, the populations passaged with heat-killed parasites evolved increased 48-hour fecundity (Fig 4). However, evolution under selection imposed by live parasites did not result in increased host fecundity or shifts in host reproductive timing, relative to the Control treatment, for either mixed mating or obligately outcrossing populations (Figs 3 and 4). Therefore, adaptive evolution was, at least in part, due to the evolution of greater defense to the parasite in both the obligately outcrossing and mixed mating populations and not the result of changes in fecundity. Thus, the mixed mating and obligately outcrossing populations followed similar evolutionary trajectories in that exposure to parasite resulted in increased host defense as opposed to general shifts in life history. Yet, host defense is a broad category and there may be multiple mechanisms driving the evolution of defense within the obligately outcrossing and mixed mating populations.

*S*. *marcescens* Sm2170 is a highly virulent parasite that can rapidly kill up to 80% of *C*. *elegans* hosts upon ingestion in naive host strains [32]. Therefore, it is most likely that selection for defense was stronger than selection for changes in fecundity in host populations. A shift in reproductive timing likely did not yield a sufficient advantage to supersede the evolution of defense in host populations exposed to such a virulent parasite. However, Control obligately outcrossing populations evolved greater 48-hour and total fecundity (Figs 3B and 4B) over the course of the experiment, while the mixed mating populations evolved greater 48-hour fecundity in the Control treatment (Fig 4A). Yet these shifts in fecundity did not result in increased fitness in the presence of the parasite for mixed mating populations and only moderately increased fitness for obligately outcrossing populations (Fig 1). Therefore, it seems that life history changes were favored by the experimental passage protocol, but not in the presence of the parasite. Rather, selection for defense may have superseded selection for changes in fecundity and reproductive timing in the presence of the parasite, regardless of the host mating system.

Different mating systems can have vastly different effects on the evolutionary trajectories of populations [36, 37]. Here, we found that obligate outcrossing increased the rate of adaptation under selection from parasites, relative to rates of adaptation in mixed mating host populations. Greater rates of adaptation in the obligately outcrossing populations may have been driven by a more rapid breakdown of linkage between beneficial and deleterious mutations [36, 37], or by more rapidly uniting beneficial mutations from different lineages [23, 24], relative to the mixed mating populations. It is possible that sexual selection was stronger on males in the obligately outcrossing populations than in the mixed mating populations, which may have also contributed to the different rates of adaptation [38–40]. However, outcrossing rates and male frequencies were maintained at high levels for several generations in the mixed mating populations exposed to parasites [10], so sexual selection is unlikely to account for the differences in the obligately outcrossing and mixed mating populations in the Evolution treatment. Conversely, male frequencies were maintained at much greater levels in the Control obligately outcrossing populations compared to the Control mixed mating populations. Therefore, sexual selection may have contributed to the increased total fecundity exhibited by the obligately outcrossing populations evolved in the absence of parasites (Fig 3B).

Overall, the host mating system did not alter the general response of the host populations. It may be that selection imposed by the parasite favoring defense was strong enough to overcome any constraints specific to mixed mating or obligate outcrossing. Additionally, the magnitude of outcrossing rates in the mixed mating populations may have been sufficient to facilitate adaptation to *S*. *marcescens* in a manner very similar to the obligately outcrossing populations. Perhaps only moderate amounts of outcrossing were necessary to facilitate the evolution of increased host defense. Indeed, our results support the prediction that mixed mating populations can gain at least some of the benefits associated with obligate outcrossing [11, 41, 42].

Although the obligately outcrossing and mixed mating populations evolved greater levels of host defense in general, many different mechanisms may contribute to these changes [1]. Resistance, tolerance, and avoidance are just three potential mechanisms that could underlie the increases in host defense. However multiple traits and many different genetic architectures may contribute to these mechanisms. Further, increased defense may have evolved through different traits in separate populations. Both behavioral [30–33] and cellular [43–45] traits are known to facilitate defense in *C*. *elegans*. Additionally, many loci are known to contribute to these traits [44–46]. Therefore, the specific traits underlying defense and the loci that contributed to the evolution of defense may have been significantly influenced by the host mating system. Nonetheless, strong selection, like that imposed by virulent parasites, may often supersede the direct effects of the host mating system on a host population’s evolutionary trajectory and promote parallel evolution. Further work is necessary to identify the components of defense in our experimental populations and more precisely determine the influence of the host mating system on their evolutionary trajectories.

## Supporting information

S1 File Fitness data. Raw competitive fitness data from the ancestral and experimental populations.
(XLS)

S2 File Host mortality data. Raw host mortality assay data from the ancestral and experimental population.
(XLS)

S3 File Host fecundity data. Raw host fecundity data over multiple days of reproduction presented as daily and total sums of offspring from ancestral and experimental populations.
(XLS)
